# Celecoxib for recurrent sclerouveitis after syphilitic panuveitis. A case report

**DOI:** 10.1007/s12348-011-0031-0

**Published:** 2011-07-22

**Authors:** Ana M. Suelves, Sebastián Martinez-Castillo, Miguel Salavert, Manuel Díaz-Llopis

**Affiliations:** 1 Department of Ophthalmology, La Nueva Fé Hospital, Valencia, Spain; 2 Department of Infectious Diseases, La Nueva Fé Hospital, Valencia, Spain; 3 Faculty of Medicine, University of Valencia, Valencia, Spain

**Keywords:** Bilateral syphilitic panuveitis, *Treponema pallidum*, Celecoxib

## Abstract

**Purpose:**

To report a case of recurrent ocular inflammation after optimal therapy of bilateral syphilitic panuveitis responding to oral celecoxib.

**Methods:**

A case report was conducted.

**Results:**

A 76-year-old man presented with painful blurry vision in both eyes. Ocular examination disclosed bilateral panuveitis. Serological testing confirmed blood and cerebrospinal fluid syphilitic involvement. After 2 weeks of intravenous penicillin therapy, recurrent episodic sclerouveitis was observed.

**Conclusion:**

Ocular inflammation after healing of infectious uveitis is a rare ophthalmic sequela. In an immunocompetent patient, either re-infection or immune uveitis should be evoked. Non-steroidal therapeutic options, as celecoxib, could be a good option of treatment in such immune cases.

## Introduction

Syphilis is experiencing an extreme increase of its incidence in Europe and the USA since the early 2000s [1]. Although penicillin is considered an excellent effective therapy against *Treponema pallidum*, patients HIV positive with cerebrospinal fluid involvement can fail treatment [2]. In immunocompetent patients, either immune uveitis or syphilitic re-infection should be evoked if ocular inflammation is observed after optimal therapy.

We report a syphilitic panuveitis presenting recurrent ocular inflammation after correct treatment with penicillin, treated with celecoxib, a nonsteroidal anti-inflammatory drug selective for cyclooxygenase-2.

## Case description

**Fig. 1:**
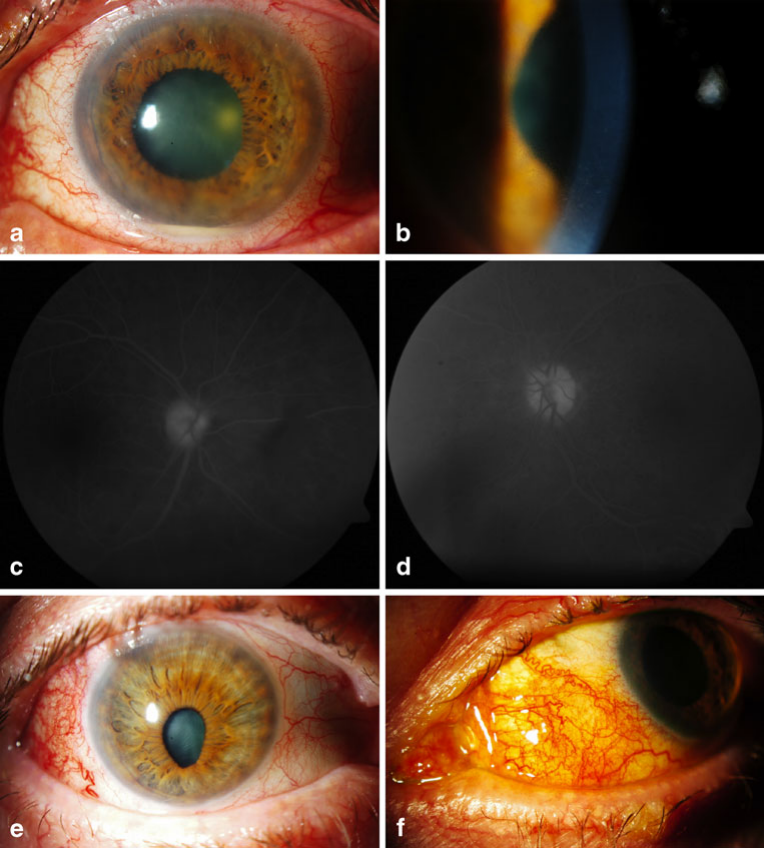
**a** Slit-lamp photograph demonstrating anterior uveitis with hypopyon in the left eye. **b** Slit-lamp photograph showing a cellular reaction and keratic precipitates in the right eye. **c** and **d** Fundus fluorescein angiogram revealing vitritis and leakage in both optic nerves. **e** and **f** Bilateral episodic anterior sclerouveitis after penicillin treatment

A 76-year-old immunocompetent man presented with discomfort and blurry vision in both eyes for the last week. He reported bilateral recurrent episodic conjunctival injection for the last year. Best-corrected visual acuity (BCVA) was 20/25 OD and 20/200 OS. Slit-lamp examination revealed 1+ cells and rare flare with fine keratic precipitates and 4+ cells with hypopyon in the right and left eye, respectively (Fig. 1a and b). Dilated fundus exam showed dense vitritis and hyperemic optic nerve with blurred margins in both eyes. Fluorescein angiography disclosed bilateral disk leakage (Fig. 1c and d). Extensive workup undertaken revealed a serum *T. pallidum* RPR titer of 1:256 and positive TPHA. Lumbar punction confirmed central nervous system involvement. No HIV infection was associated. The patient completed a 2-week cycle of intravenous penicillin G and topical steroids, experiencing a complete resolution of panuveitis and BCVA of 20/20 in both eyes. After 3 weeks of the end of treatment, the patient presented up to three anterior sclerouveitis episodes, responding to topical steroids. A re-infection of *Treponema* was microbiologically and clinically ruled out, so an immune ocular response was evoked, starting with celecoxib 200 mg a day, with progressive improvement and no recurrences observed for 1 year of follow-up.

## Discussion

Interaction of *T. pallidum* with the host's immune system stimulates humoral and cellular immunity. *T. pallidum* produces lipoproteins on the outer membrane that interact with lipopolysaccharide-binding proteins, inducing the expression of inflammatory mediators via CD14 and toll-like receptor 2 recognition. Treponemes also possess glycolipids linked to the outer membrane that resemble bacterial lipopolysaccharide. These glycolipids induce cellular activation by releasing pro-inflammatory cytokines, mainly tumoral necrosis factor alpha [3], even after successful penicillin treatment.

Recurrent ocular inflammation after infectious uveitis treatment is a rare ocular sequela. In the immunocompetent host, such as our patient, it can occur due to a syphilitic re-infection or to an immune process, which may be caused by antigenically inert treponema cell surface. To our knowledge, this is the first report of an immune response after optimal treatment of ocular syphilis at an immunocompetent host.

NSAIDs have an anti-chemotactic activity and modulate both humoral and cellular pathways. Systemic NSAIDs such celecoxib are known to control ocular inflammatory processes such chronic iridocyclitis or recurrent acute anterior uveitis [4, 5], being suitable and sparing-steroids option of therapy in these cases.

In conclusion, the outer treponema surface may play a role in immune responses observed after syphilis treatment. We present the first case reported in literature of immune sclerouveitis after neurosyphilis treatment treated successfully with celecoxib.
